# Contribution of cuproptosis and immune-related genes to idiopathic pulmonary fibrosis disease

**DOI:** 10.3389/fimmu.2025.1458341

**Published:** 2025-02-07

**Authors:** Chengji Jin, Jia Li, Qiaoyu Li, Lipeng Zhang, Shaomao Zheng, Qiong Feng, Yongjie Li, Yu Zheng, Qiuli Nie, Jin Liang, Jing Wang

**Affiliations:** ^1^ Department of Respiratory Medicine, The Second Affiliated Hospital, Hainan Medical University, Haikou, China; ^2^ The Second School of Clinical Medicine, Hainan Medical University, Haikou, China; ^3^ Department of Thoracic Surgery, The Second Affiliated Hospital, Hainan Medical University, Haikou, China; ^4^ Department of Rheumatology and Immunology, The Second Affiliated Hospital, Hainan Medical University, Haikou, China; ^5^ National Health Commission (NHC) Key Laboratory of Tropical Disease Control, Hainan Medical University, Haikou, China

**Keywords:** idiopathic pulmonary fibrosis disease, cuproptosis -related genes, immune-related genes, immune infiltration, single-cell RNA-seq

## Abstract

**Background:**

Idiopathic pulmonary fibrosis (IPF) is a degenerative respiratory condition characterized by significant mortality rates and a scarcity of available treatment alternatives. Cuproptosis, a novel form of copper-induced cell death, has garnered attention for its potential implications. The study aimed to explore the diagnostic value of cuproptosis-related hub genes in patients with IPF. Additionally, multiple bioinformatics analyses were employed to identify immune-related biomarkers associated with the diagnosis of IPF, offering valuable insights for future treatment strategies.

**Methods:**

Four microarray datasets were selected from the Gene Expression Omnibus (GEO) collection for screening. Differentially expressed genes (DEGs) associated with IPF were analyzed. Additionally, weighted gene coexpression network analysis (WGCNA) was employed to identify the DEGs most associated with IPF. Ultimately, we analyzed five cuproptosis-related hub genes and assessed their diagnostic value for IPF in both the training and validation sets. Additionally, four immune-related hub genes were screened using a protein–protein interaction (PPI) network and evaluated through the receiver operating characteristic (ROC) curve. Lastly, single-cell RNA-seq was employed to further investigate differential gene distribution.

**Results:**

We identified a total of 92 DEGs. Bioinformatics analysis highlighted five cuproptosis-related genes as candidate biomarkers, including three upregulated genes (*CFH*, *STEAP1*, and *HDC*) and two downregulated genes (*NUDT16* and *FMO5*). The diagnostic accuracy of these five genes in the cohort was confirmed to be reliable. Additionally, we identified four immune-related hub genes that demonstrated strong diagnostic performance for IPF, with *CXCL12* showing an AUROC of 0.90. We also examined the relationship between these four genes and immune cells. *CXCL12* was significantly negatively associated with neutrophils, while *CXCR2* was associated exclusively with neutrophils, consistent with our single-cell sequencing results. *CTSG* showed a primarily positive association with follicular helper T, and *SPP1* was most strongly associated with macrophages. Finally, our single-cell sequencing data revealed that in patients with IPF, *CXCL12* was highly expressed in the endothelial cell subset (ECs), while *SPP1* exhibited high expression in multiple cellular populations. The expression of the *CTSG* showed statistically significant differences in monocyte macrophages.

**Conclusion:**

The research methodically depicted the intricate interplay among five cuproptosis-related genes, four immune-related hub genes, and IPF, offering new ideas for diagnosing and treating patients with IPF.

## Introduction

Idiopathic pulmonary fibrosis (IPF) represents a long-term, advancing pulmonary condition with scarce therapeutic choices, high mortality, and poor prognosis, with a median survival of only 2.5–3.5 years from the time of diagnosis (1–3). Epidemiologic surveys show that the global incidence of IPF ranges from 0.09 to 1.30 per 10,000 people, with an increasing trend over the years (4). At present, the pathophysiological mechanism of IPF has not been fully elucidated. It has been suggested that the occurrence of IPF may be closely related to persistent or repetitive injury to alveolar epithelial cells. Dysregulated epithelial cells interact with mesenchymal cells, immune cells, and endothelial cells through various signaling mechanisms, which contribute to tissue scarring, modification of the alveolar structure, and irreversible loss of lung function (5). Immune cells play a pivotal role in the onset and progression of fibrogenesis by promoting or exacerbating tissue structural remodeling (6, 7). Continued damage to alveolar epithelial cells leads to an increase in neutrophils and monocytes, triggering inflammatory responses (8). Similarly, macrophages are crucial in the initiation and progression of IPF (9). In addition, the wound healing process involves an inflammatory response that recruits fibroblasts, activates myofibroblasts, and deposits extracellular matrix in the form of collagen and other proteins (10). Although pirfenidone and nintedanib are recommended in the guidelines, their efficacy is limited. Therefore, further exploration of novel therapeutic strategies for IPF is particularly important (11).

Copper is one of the essential trace elements in the human body and plays a key role in many biological processes (12). It is involved in multiple physiological processes, including the regulation of energy metabolism, mitochondrial respiration, and antioxidant activity (13–15). Copper ion levels maintain a dynamic equilibrium, and a loss of this balance can lead to oxidative stress and abnormal cellular autophagy (16, 17), causing a variety of copper or copper ion-related diseases. Cuproptosis is a recently discovered, unique form of cell death. Upon disruption of the mitochondrial respiratory chain, the lipoylated components of the tricarboxylic acid cycle are directly bound by copper ions, forming aggregates that ultimately lead to cell death (13). Furthermore, to date, no studies have demonstrated a relationship between cuproptosis and fibrosis. Previous studies have shown that the signaling pathway mediated by the fibrotic cytokine transforming growth factor β1 (TGF-β1) plays a significant role in lung fibrosis. TGF-β1 reduces Nicotinamide adenine dinucleotide (NADH) and NADH/NAD levels, possibly due to alterations in the tricarboxylic acid cycle, which results in decreased ATP levels and impaired oxidative phosphorylation in lung fibroblasts (18). Therefore, we hypothesized that cuproptosis may play a role in the development of IPF. Genes associated with cuproptosis could serve as new targets for IPF therapy, although their precise mechanisms remain to be elucidated. In this study, we conducted bioinformatics analyses to identify novel biomarkers of IPF that may be associated with cuproptosis and immunity.

## Materials and methods

### Collection and data processing of microarray datasets for IPF

**Figure 1:**
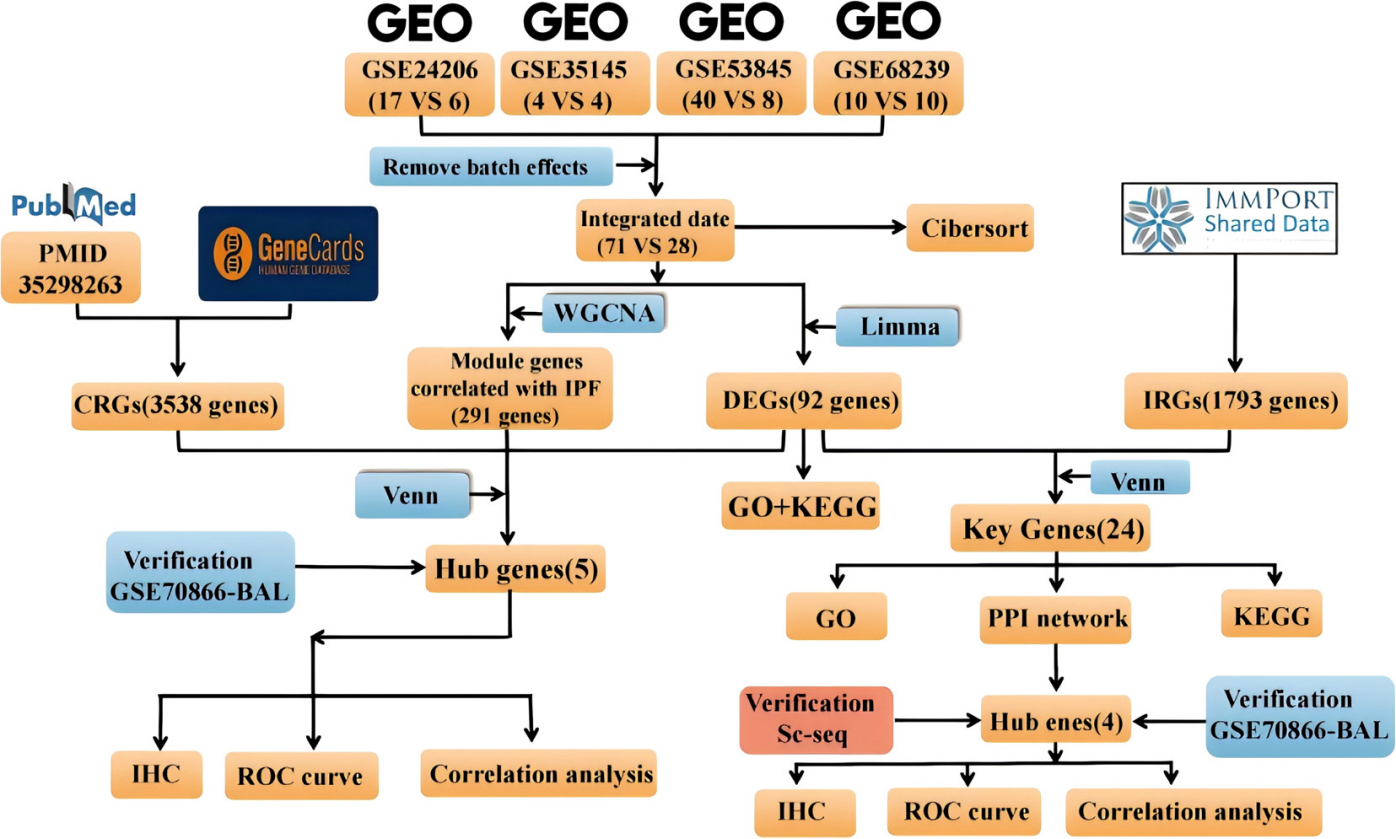
Overview of the multistep approach employed in our systemic analysis.

The study flow diagram is shown in **Figure 1**. Five datasets were obtained from the Gene Expression Omnibus database (https://www.ncbi.nlm.nih.gov/geo/). Specifically, GSE24206, GSE35145, GSE53845, and GSE68239 were utilized as the training sets, while GSE70866 served as the validation set. Initially, the four training sets were combined using the R package inSilicoMerging (19). Subsequently, the method proposed by Johnson et al. (20) was applied to eliminate batch effects, resulting in a unified Gene Expression Omnibus (GEO) dataset consisting of 99 samples from 71 IPF patients and 28 normal controls. This unified dataset was then utilized to identify differentially expressed genes (DEGs). In this study, differential analysis was performed using the R package limma (version 3.40.6) to identify genes that exhibit differential expression between various control groups. Genes meeting the criteria of an adjusted *p*-value < 0.05 and |log2 fold change (FC)| > 1.5 were classified as DEGs. The volcano plot and heatmap displayed the expression data of 92 DEGs. Additionally, the density plot and Uniform Manifold Approximation and Projection (UMAP) plot results indicated the successful elimination of batch effects. The microarray preprocessing outcomes were visualized through a boxplot. Gene ontology (GO) enrichment analysis and Kyoto Encyclopedia of Genes and Genomes (KEGG) pathway analysis were conducted using the org.Hs.eg.db (version 3.1.0) and “clusterProfiler” (version 3.14.3) package in R to explore the biological roles of DEGs. The GO annotation and KEGG pathway analysis were performed with a significance threshold of *p* < 0.05.

### Assessment of immune cell infiltration

The development of IPF is closely linked to the immune system, and an immune response plays a crucial role at every stage of fibrosis (21). The CIBERSORT tool was used to explore the differences in the proportions of 22 immunocyte types between the IPF group and the controls (22). Using a known reference set, this approach generates a set of gene expression profiles for 22 immune cell subtypes.

### Construction of WGCNA coexpression network

Using gene expression patterns, we first calculated Media Absolute Deviation (MAD) for each gene, removed the top 50% of genes with the smallest MAD values, and excluded outlier genes and samples using the weighted gene coexpression network analysis (WGCNA) GoodSamplesGenes approach in the R package. We then constructed a scale-free coexpression network using WGCNA, setting the soft-threshold power to 4. To further analyze the module, we calculated the dissimilarity of module eigengenes, selected a cut line for the module dendrogram, and merged certain modules. In addition, we merged modules with distances less than 0.25, resulting in 11 coexpression modules. The gray module was identified as a collection of genes that could not be assigned to a singular module. Among these, the yellow module was found to be the most relevant for subsequent research projects.

### Acquisition of differentially expressed genes associated with cuproptosis-related pathways

A total of 2,181 cuproptosis-related genes (CRGs) were identified in GeneCard (https://www.genecards.org), and genes with correlation coefficients above the median were selected, resulting in 1,090 genes. Additionally, following the guidelines of Tsvetkov et al. (13), we identified another 2,978 genes associated with cuproptosis. After combining the two gene sets to remove duplicates, a total of 3,538 CRGs were selected for subsequent analysis in this article.

### Identification of DEIRGs and establishment of PPI network

A compilation of immune-related (IRGs) was obtained from the Immunology Database and Analysis Portal (ImmPort, https://www.immport.org/shared/genelists). A total of 24 differently expressed immune-related genes (DEIRGs) were identified by intersecting the previously obtained DEGs with IRGs.

Protein–protein interaction (PPI) network was constructed using the STRING database (http://string-db.org) to identify key genes. Cytoscape software (version 3.9.1) was utilized to visualize the PPI networks, and the Cytoscape plugin CytoNCA was employed to identify genes associated with hub genes.

### Immunohistochemistry

Each sample was fixed in a 4% neutral formaldehyde solution and embedded in paraffin wax. The tissue was sectioned into 4 µM slices, which were then dewaxed and rehydrated. Initially, the slices were placed in a preheated repair solution at 65°C, heated to 90°C for a 30-min incubation, and subsequently cooled to 70°C. Following this, the slices were washed with PBS and incubated with hydrogen peroxide for 10 min. The primary antibodies used for the analysis included *STEAP1* (bs-1901R, Bioss, Beijing, China), *CFH* (bs-9525R, Bioss, Beijing, China), *HDC* (bs-1054R, Bioss, Beijing, China), *FMO5* (bs13187R, Bioss, Beijing, China), *CXCL12* (bs-4938R, Bioss, Beijing, China), *CXCR2* (abs-133162, Absin, Shanghai, China), and *SPP1* (AF0227, Affinity, Jiangsu, China). The primary antibody reactions were carried out for 30 min, followed by a 20-min reaction with the secondary antibody (PV-6000, OriGene, Wuxi, China). The sections were counterstained with hematoxylin, dehydrated, cleared, and sealed according to conventional protocols. Images were then captured under a light microscope, and cells stained brown were considered positive.

### Lung specimen preparation

This study included eight human lung specimens, comprising three from healthy controls and five from patients with IPF. The healthy control specimens were derived from residual biopsy specimens deemed unsuitable for lung transplantation, while the IPF samples were obtained during lung transplantation procedures. The inclusion criteria for IPF patients were based on the diagnostic standards set by the American Thoracic Society/European Respiratory Society. All participants provided written informed consent after receiving a comprehensive explanation of the study.

### Tissue dissociated single cell suspension

Pulmonary nonparenchymal cells were isolated from fresh lung specimens. Lung samples were sliced into 1–2 mm sheets and incubated in 2 ml of GEXSCOPE^®^ Tissue Dissociation Solution at 37°C for 15 min to maintain warmth. The resulting single-cell mixture was dispensed onto an array and incubated for an additional 15 min. After digestion, filter the sample through a 40 µm sterile mesh filter, and centrifuge the filtrate at 1000 rpm for 5 minutes. Discard the supernatant and resuspend the cells with 1 ml of PBS (HyClone). Add 2 mL of GEXSCOP® Erythrocyte Lysis Buffer (Singleron, Nanjing, China) and allow to stand for 10 minutes at 25°C. Centrifuge at 500 g for 5 minutes, and resuspend the cells using 1 ml of PBS. Then stain with Trypan Blue (Sigma) and count the viable cells and total cells under a microscope.

### Sequencing of single-cell transcriptome libraries

Single-cell suspensions preserved in PBS at a concentration of 1 × 105 cells/ml were prepared and loaded onto a microfluidic chip. scRNA-seq libraries were constructed using the GEXSCOPE^®^ Single-Cell RNA Library Kit (Singleron Biotechnologies, Nanjing, China) in accordance with the Singleron GEXSCOP^®^ Operating Instructions. The libraries were then diluted to 4 nM and sequenced on the Illumina Novaseq6000 sequencing platform using 150 bp paired-end sequencing mode.

### Quality control and analysis of single-cell sequencing data

Raw sequencing data obtained from the sequencing run were processed using Singleron’s internal analysis pipeline to generate a gene expression matrix. Briefly, reads1 without poly-T sequences were filtered out, and valid cell barcodes and UMIs were extracted. Reads2 were filtered to remove adapters and poly-A tails (using fastp V1). The data were then aligned and quantified against the reference genome from the Ensembl database using STAR (v2.5.3a) and featureCounts (v1.6.2). The pipeline group reads, UMIs, and genes sharing the same cell barcode and calculates the number of UMIs for each gene in every cell for subsequent analysis. The Seurat package (version 3.0.1) was used for cell type identification and clustering analysis of the RNA sequencing data. The expression matrix was imported into R using the read.table function, and cell clustering analysis was performed using the FindClusters function (with a resolution parameter set to 0.6). DEGs between different samples or continuous clusters were identified using the findMarkers function.

The study was reviewed and approved by the Ethics Committee of The Second Affiliated Hospital of Hainan Medical University.

### Statistical analysis

SPSS 22.0 was used to process and statistically analyze the data. *p*-values of less than 0.05 were taken as the criterion for statistical significance. Comparisons of continuous variables were verified using the Student’s *t*-test Kruskal–Wallis *H* test. The Chi-square test or Fisher’s exact test was used for categorical variables. Associations between pivotal genes and immune cells were analyzed using Spearman’s rank sum test or Pearson’s correlation coefficient. Receiver operating characteristic (ROC) analysis was performed using the R package pROC (version 1.17.0.1) to determine the diagnostic accuracy of the hub genes. The results were expressed as the area under the ROC curve (AUROC) with 95% CI. AUROC ≥ 0.9 indicates exceptional discrimination, 0.8 ≤ AUROC < 0.9 indicates excellent discrimination, 0.7 ≤ AUROC < 0.8 indicates acceptable discrimination, 0.5 ≤ AUROC < 0.7 indicates limited diagnostic effectiveness, and ROC = 0.5 indicates no discrimination). Spearman’s rank test or Pearson’s correlation coefficient was used to analyze the associations between hub genes and immune cells.

## Results

### GEO dataset collection and preprocessing

**Figure 2:**
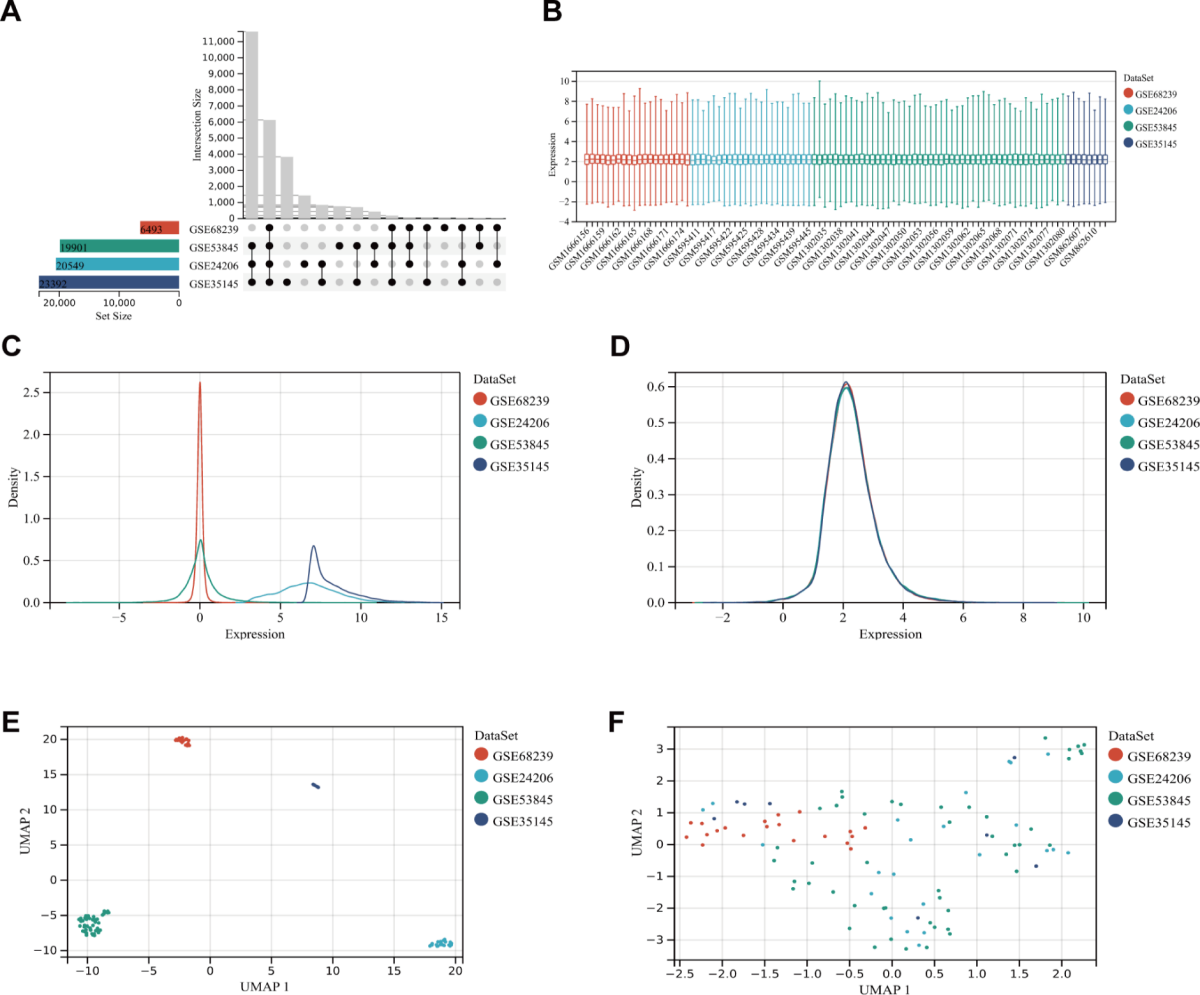
DEG dataset collection and pre-processing. **(A)** Detailed information about the collected datasets. **(B)** Boxplot of normalized microarray data. **(C, D)** Density plots showing differences in sample distribution before and after debatching. **(E, F)** UMAP visualizations illustrating how samples clustered before and after the removal of the batch effect.

After filtering the GEO microarray datasets, we retained four datasets: GSE24206, GSE35145, GSE53845, and GSE68239 (**Figure 2A**). These were merged to create an internal dataset. The box plots illustrate the distribution of differences and confirm the standardization of the internal datasets (**Figure 2B**). The results from the primary density studies indicate a high level of consistency in the internal dataset after excluding batch effects (**Figures 2C, D**). Furthermore, the UMAP plot demonstrates a convergence of data distribution across datasets following the elimination of batch effects (**Figures 2E, F**).

### Identification of DEGs and functional and pathway enrichment analysis of DEGs

**Figure 3:**
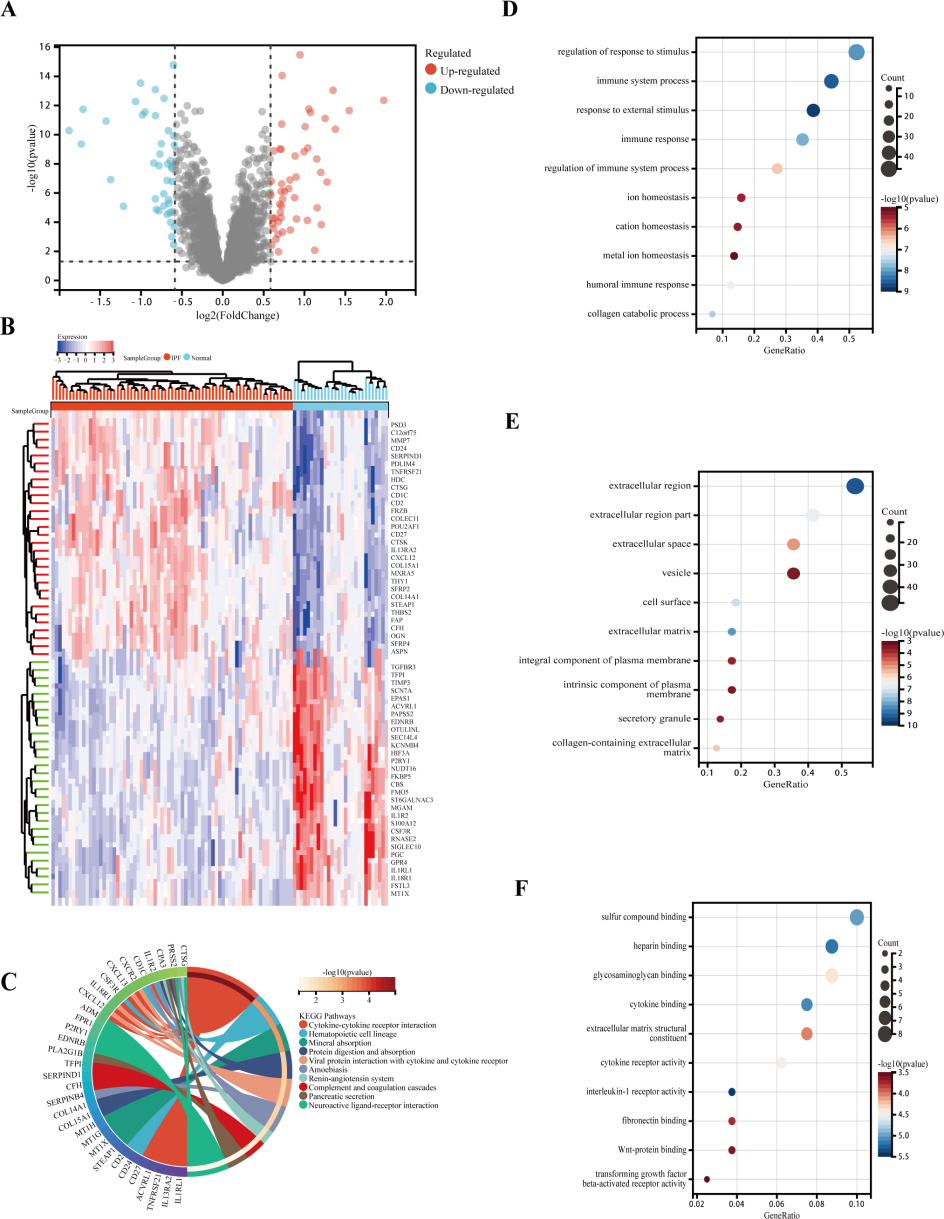
Functional and pathway enrichment analysis of DEGs. **(A)** Volcano plot of DEGs: blue nodes represent downregulation in IPF, red nodes represent upregulation, and gray nodes represent no significant difference from controls. **(B)** Heatmap of 92 IPF-related DEGs. **(C)** Gene ontology (GO) molecular function pathway. **(D)** GO biological processes pathway. **(E)** GO cellular component pathway. **(F)** GO molecular function pathway.

DEGs were analyzed in RNA samples from IPF and normal controls using the “limma” software package, identifying a total of 92 DEGs, with 41 downregulated and 51 upregulated genes. A volcanic graph was generated using a 1.5-fold change criterion (**Figure 3A**). **Figure 3B** illustrates the heatmap for DEGs. To investigate the potential functions of these genes, GO and KEGG enrichment pathway analyses were conducted using the R clusterProfiler package. The KEGG analysis revealed that the DEGs were enriched in the following pathways: cytokine–cytokine receptor interaction, hematopoietic cell lineage, and mineral absorption (**Figure 3C**). The GO-BP analysis (**Figure 3D**) showed significant enrichment in the immune system process, immune response, metal ion homeostasis, and cation homeostasis. In the GO-CC analysis (**Figure 3E**), we observed enrichment in the extracellular matrix, cell surface, and extracellular space. Furthermore, the GO-MF enrichment analysis (**Figure 3F**) revealed fibronectin binding, Wnt-protein binding, interleukin-1 receptor activity, and transforming growth factor beta-activated receptor activity. These findings suggest that the DEGs play a crucial role in IPF and warrant further investigation.

### Assessment of immune cell infiltration

**Figure 4:**
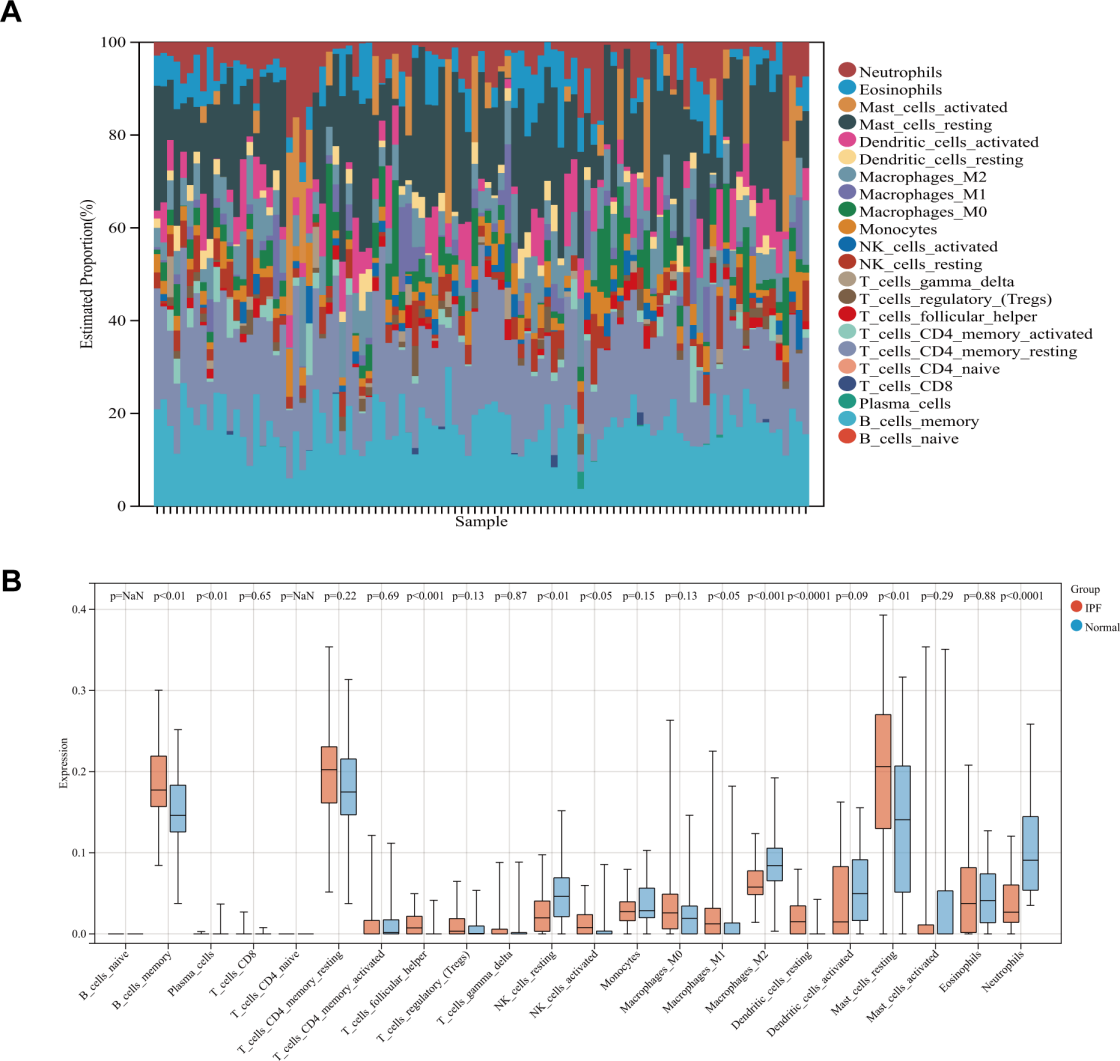
**(A)** Stacked histogram comparing immune cell percentages between IPF and control samples. **(B)** Boxplot showing the abundance of 22 immune cell types.

Since immune cells play a key role in the development and initiation of fibrosis, we analyzed immune cell infiltration. Layered histograms display the abundance distribution of 22 types of immune cells across each sample (**Figure 4A**). Different immune cell types are represented by distinct colors, with the height of each color indicating the proportion of cells. **Figure 4B** highlights a significant difference in the expression levels of 10 different immune cell marker types. Six immune cell markers (memory B cells, follicular helper T cells, activated NK cells, M1 macrophages, resting dendritic cells, and resting mast cells) were significantly upregulated in the IPF group. Concurrently, the expression levels of plasma cells, dormant NK cells, M1 macrophages, and neutrophils were reduced compared to the control group.

### Construction of WGCNA

**Figure 5:**
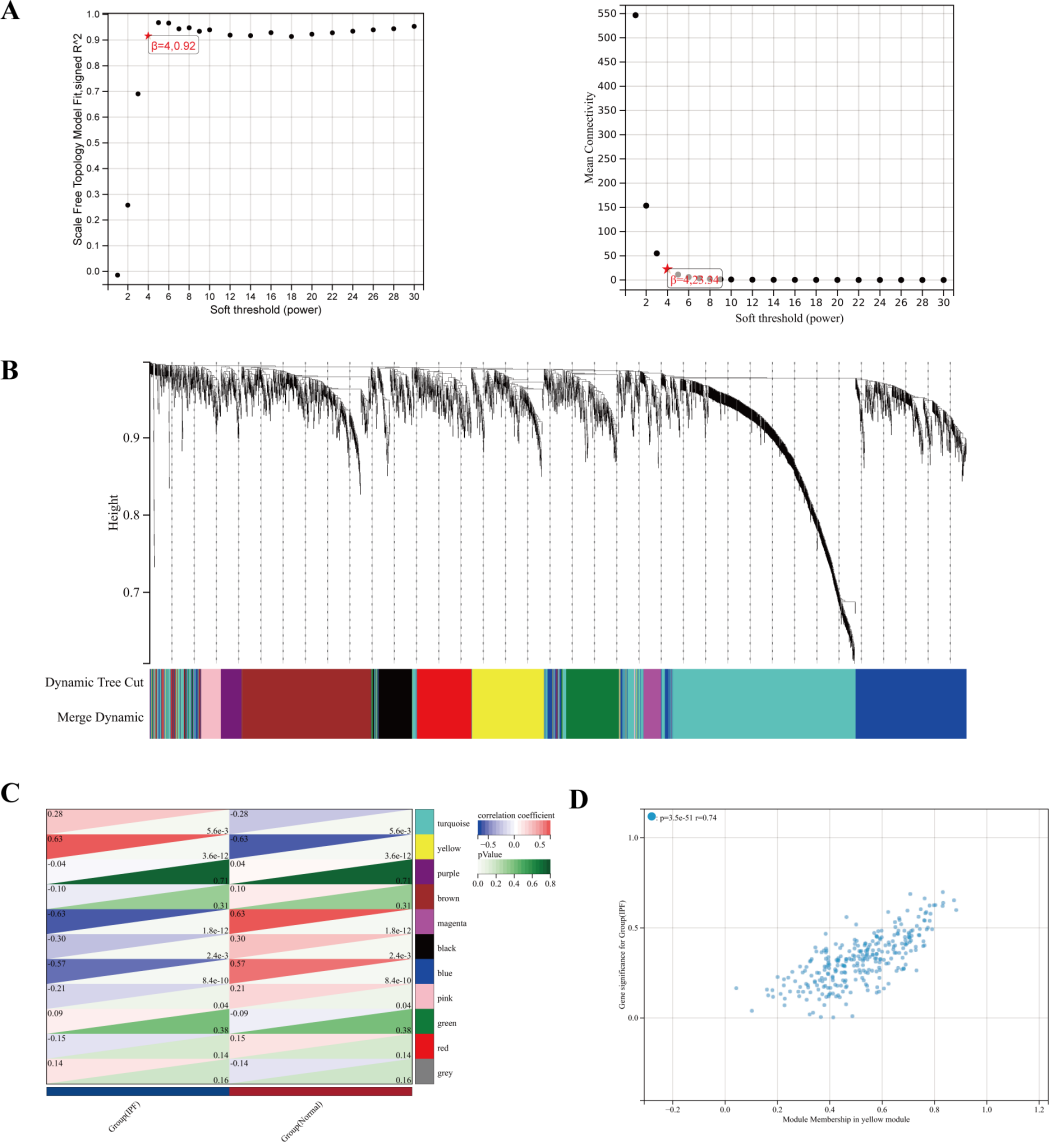
**(A)** The power index of 4 was chosen as the appropriate soft threshold, achieving a scale-free coexpression network. **(B)** The branches of the dendrogram correspond to 11 gene modules. **(C)** The correlation coefficients and corresponding *p*-values between each module and IPF. **(D)** Scatter plot of module eigengenes in the yellow module.

To study the key genes in depth, we selected a soft threshold of 4 (**Figure 5A**), developed a gene coexpression network utilizing WGCNA technology, and identified the modules most closely related to IPF (**Figure 5B**). In total, 11 gene modules were obtained, with the yellow module showing a significant correlation with IPF (correlation coefficient = 0.63, *p* < 0.001) (**Figure 5C**). The yellow module, identified as the key module, contains 291 genes. **Figure 5D** displays the scatter plot of module eigengenes in the yellow module.

### Identification of cuproptosis-related hub genes and performance in training-focused diagnostics of IPF

We identified 2,978 CRGs from a previous study (13). A total of 2,181 CRGs were extracted from GeneCards, and 1,090 genes were obtained after taking the median number. After merging the two genes and removing duplicates, we ultimately ended up with 3,538 CRGs.

**Figure 6:**
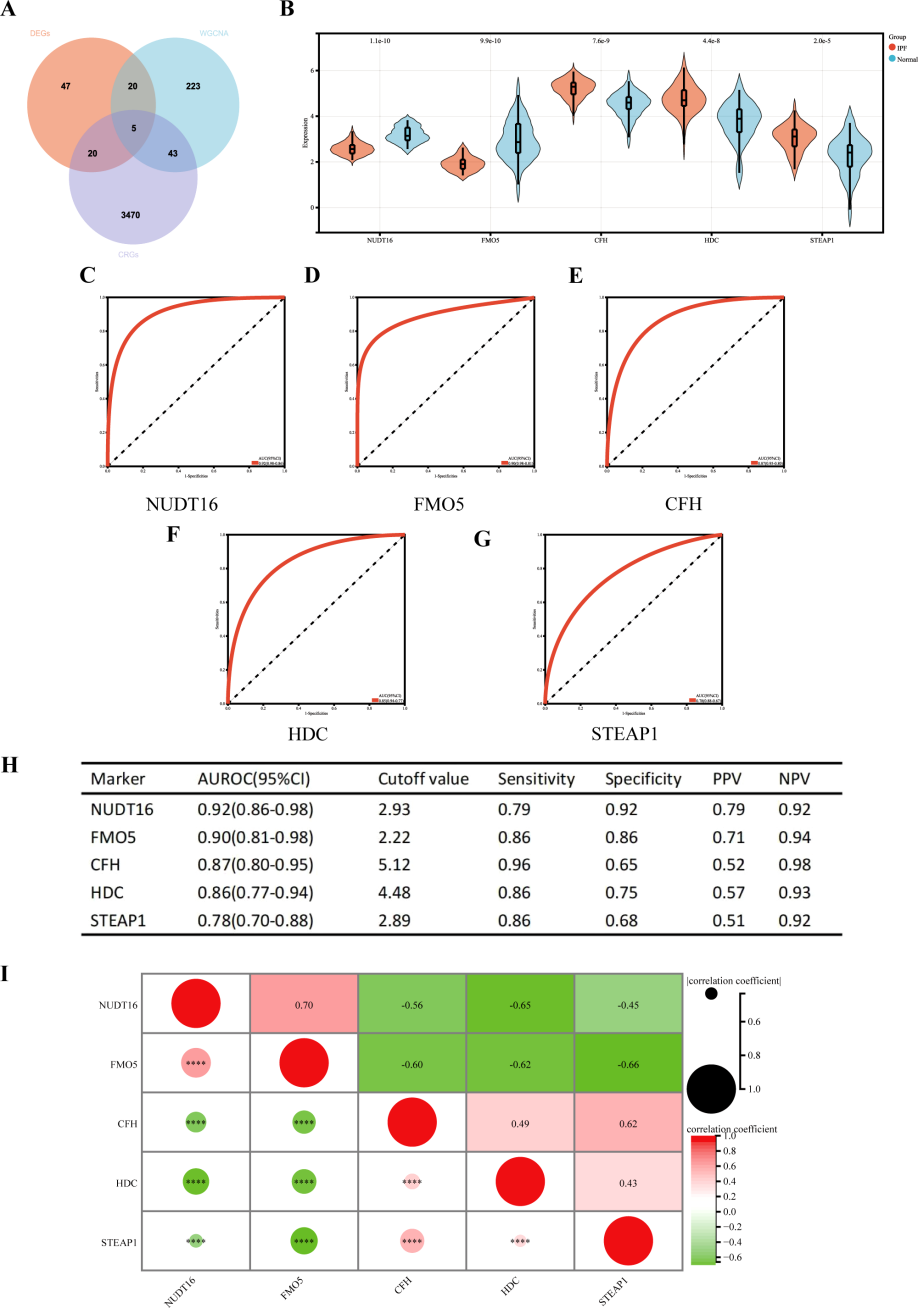
**(A)** Venn diagram showing the intersection of diagnostic markers obtained from the three algorithms. Performance of the five hub genes in diagnosing IPF in the validation set. **(B)** Expression differences of the five hub genes in IPF and control groups. **(C–G)** ROC curves of the five hub genes in IPF and control groups. **(H)** Diagnostic value of the five hub genes for differentiating between IPF and control groups. **(I)** Correlation between the five hub genes. PPV, positive predictive value; NPV, negative predictive value; AUROC, area under the receiver operating characteristic curve. ^****^ *p* < 0.0001.

Five cuproptosis-related hub genes (*NUDT16*, *FMO5*, *CFH*, *HDC*, and *STEAP1*) were identified by crossing the candidate genes obtained from the DEGs, CRGs, and WGCNA models (**Figure 6A**). **Figure 6B** displays the comprehensive expression patterns of the hub genes when comparing IPF samples with normal samples. *CFH*, *HDC*, and *STEAP1* exhibited prominent expression in IPF, while the expression of *NUDT16* and *FMO5* was reduced in the IPF group (all *p* < 0.001). The ROC curve showed that the AUROC for *NUDT16* was 0.92 (95% CI = 0.86–0.98), with sensitivity and specificity of 0.79 and 0.92 (**Figures 6C, H**). For *FMO5*, the AUROC was 0.90 (95% CI = 0.81–0.98), and the sensitivity and specificity were 0.86 and 0.86 (**Figures 6D, H**). The AUROC for *CFH* in the diagnosis of IPF was 0.87 (95% CI = 0.80–0.95), with sensitivity and specificity of 0.96 and 0.65 (**Figures 6E, H**). The AUROC of *HDC* was 0.86 (95% CI = 0.77–0.94), with sensitivity and specificity of 0.86 and 0.75 (**Figures 6F, H**). The sensitivity, specificity, and AUROC of *STEAP1* were 0.86, 0.68, and 0.78 (95% CI = 0.70–0.88), respectively (**Figures 6G, H**).

We also performed a correlation analysis of the five hub genes (**Figure 6I**). The results showed that *NUDT16* was only positively correlated with *FMO5* (*r* = 0.70, *p* < 0.001), significantly negatively correlated with *HDC* (*r* = 0.65, *p* < 0.001), and moderately negatively correlated with *CFH* (*r* = 0.56, *p* < 0.001) and *STEAP1* (*r* = 0.45, *p* < 0.001). *FMO5* was negatively correlated with *CFH* (*r* = 0.60, *p* < 0.001), HDC (*r* = 0.62, *p* < 0.001), and *STEAP1* (*r* = 0.66, *p* < 0.001). *HDC* was moderately positively associated with *STEAP1* (*r* = 0.43, *p* < 0.001). In summary, the results suggest that most of the five genes are highly correlated.

### Performance of hub genes in diagnosing IPF in the validation set and the results of immunohistochemistry

**Figure 7:**
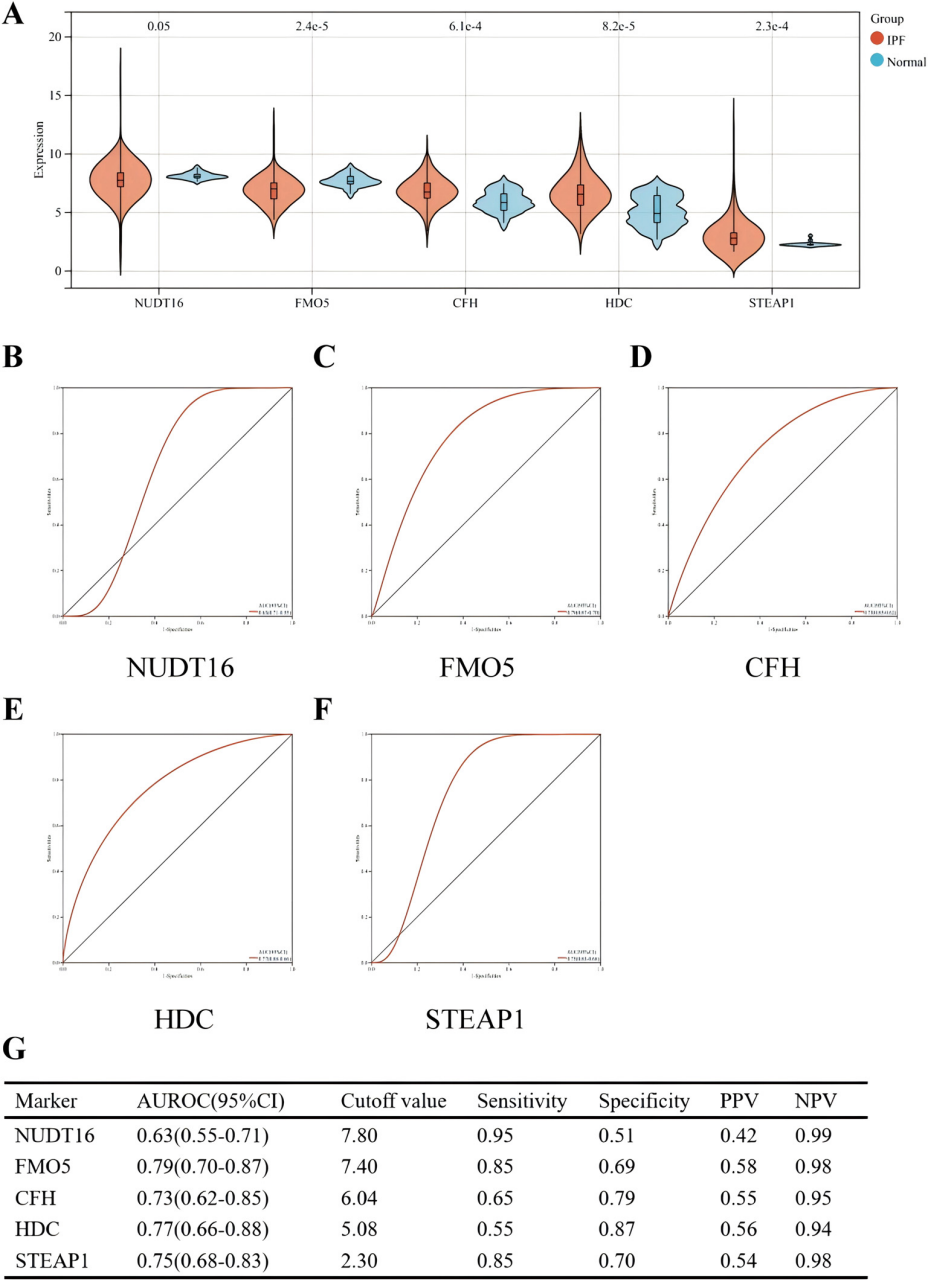
Performance of the five hub genes in diagnosing IPF in the validation set. **(A)** Expression differences of the five hub genes between IPF and control groups. **(B–F)** ROC curves of the five hub genes in IPF and control groups. **(G)** Diagnostic value of the four hub genes for differentiating between IPF and control groups. PPV, positive predictive value; NPV, negative predictive value; AUROC, area under the receiver operating characteristic curve.

The diagnostic efficacy of the five genes in the validation set (GSE70866) was also excellent (**Figure 7**). The validation group consisted of alveolar lavage fluid specimens, with 196 specimens in total, including 176 from patients with IPF. **Figure 7A** shows that *CFH*, *HDC*, and *STEAP1* (all *p* < 0.001) were significantly overexpressed in IPF, with AUROC of 0.73 (95% CI = 0.62–0.85), 0.77 (95% CI = 0.66–0.88), and 0.75 (95% CI = 0.68–0.83), respectively (**Figures 7D, F**). However, the expressions of *NUDT16* (*p* = 0.05) and *FMO5* (*p* < 0.001) were significantly lower in the IPF group compared to controls. The AUROC for these genes were 0.63 (95% CI = 0.55–0.71) and 0.79 (95% CI = 0.70–0.87) (**Figures 7B, C**), which is consistent with the expression we obtained earlier.

**Figure 8:**
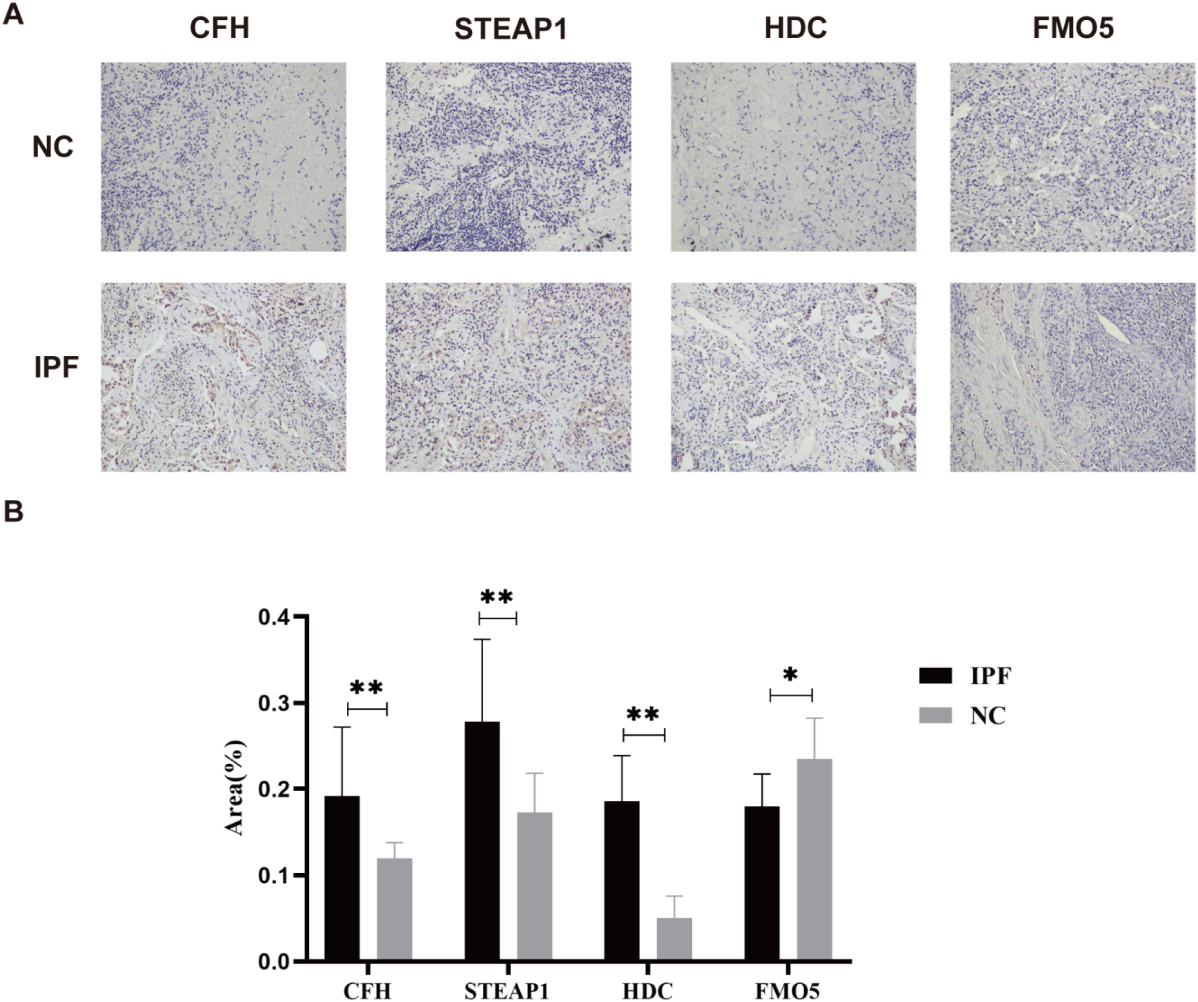
**(A)** Representative images of immunohistochemical staining of *CFH*, *STEAP1*, *HDC*, and *FMO5* in lung tissues. **(B)** The areas of *CFH*, *STEAP1*, *HDC*, and *FMO5*. ^*^ *p* < 0.05; ^**^ *p* < 0.001.

Not coincidentally, immunohistochemistry of IPF and normal tissues also demonstrated similar results. The immunohistochemistry (IHC) analysis revealed positive expression of *CFH*, *STEAP1*, and *HDC* in the IPF group (all *p* < 0.01), while *FMO5* was overexpressed in the control group (*p* < 0.05) (**Figures 8A, B**). Unfortunately, we did not detect *NUDT16* expression.

### Analysis focusing on the functionality and pathway enhancement of differently expressed immune-related genes

**Figure 9:**
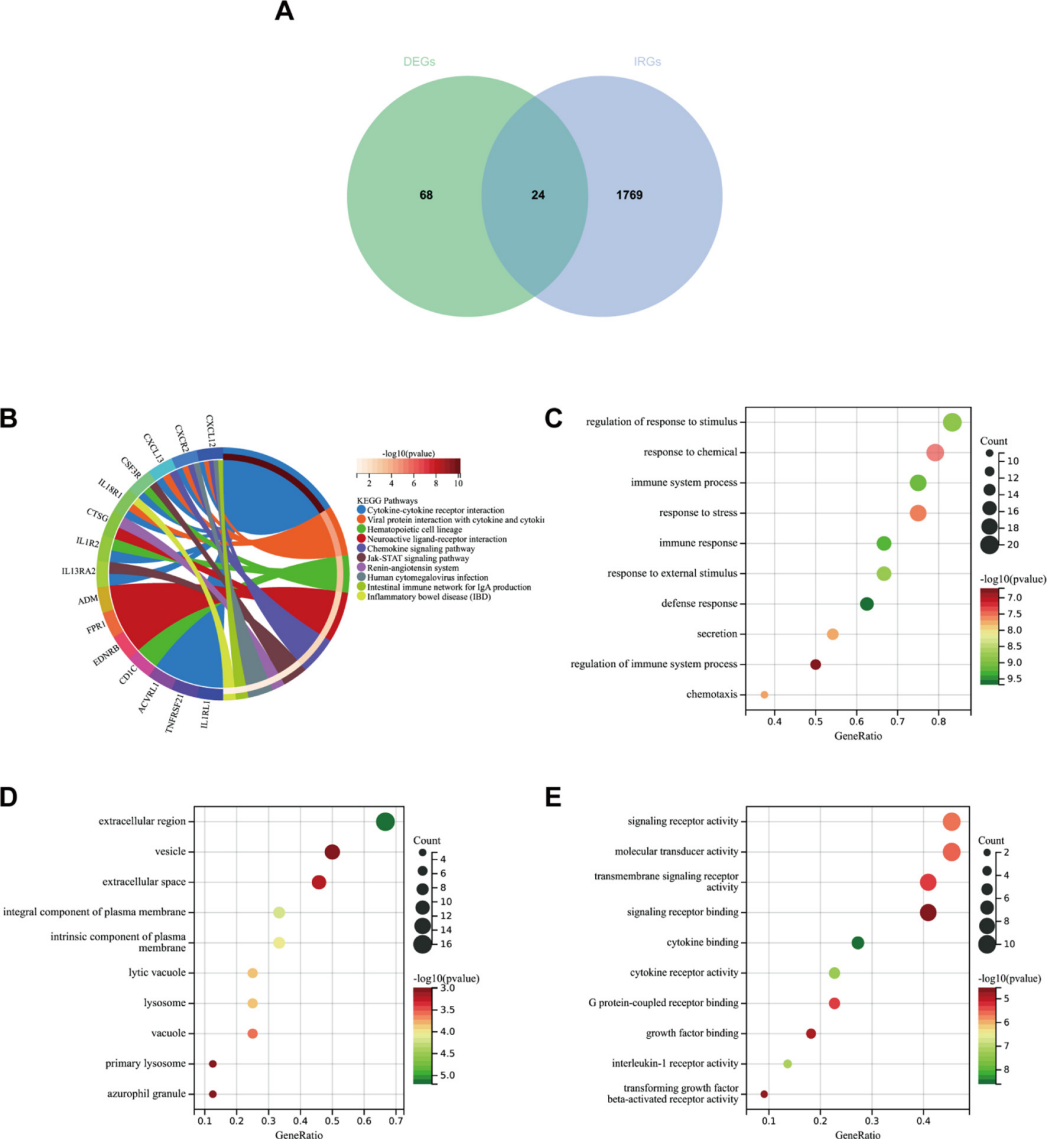
**(A)** Venn diagram showing the intersection of diagnostic markers obtained from DEGs and IRGs. **(B)** Top 10 KEGG pathways. **(C)** Top 10 gene ontology (GO) biological process pathways. **(D)** Top 10 GO cellular component pathways. **(E)** Top 10 GO molecular function pathways.

Firstly, we extracted 1,793 immune genes associated with IPF from the “Immport Shared Data”. As shown in **Figure 9A**, 24 DEIRGs were screened after intersecting the 92 DEGs with the 1,793 IRGs. In the KEGG analysis (**Figure 9B**), the most enriched pathways were cytokine–cytokine receptor interaction, neuroactive and ligand–receptor interaction, and viral protein interaction with cytokine–cytokine receptor, among others.

In GO-BP analysis (**Figure 9C**), the major pathways were regulation of response to stimulus, defense response, and immune response. The results of enrichment analysis in GO-CC (**Figure 9D**) revealed enrichment in the extracellular region, an integral component of the plasma membrane, and others. GO-MF analysis (**Figure 9E**) indicated that the major pathways included signaling receptor activity and cytokine binding, among others.

### Construction of PPI network and screening of hub genes

**Figure 10:**
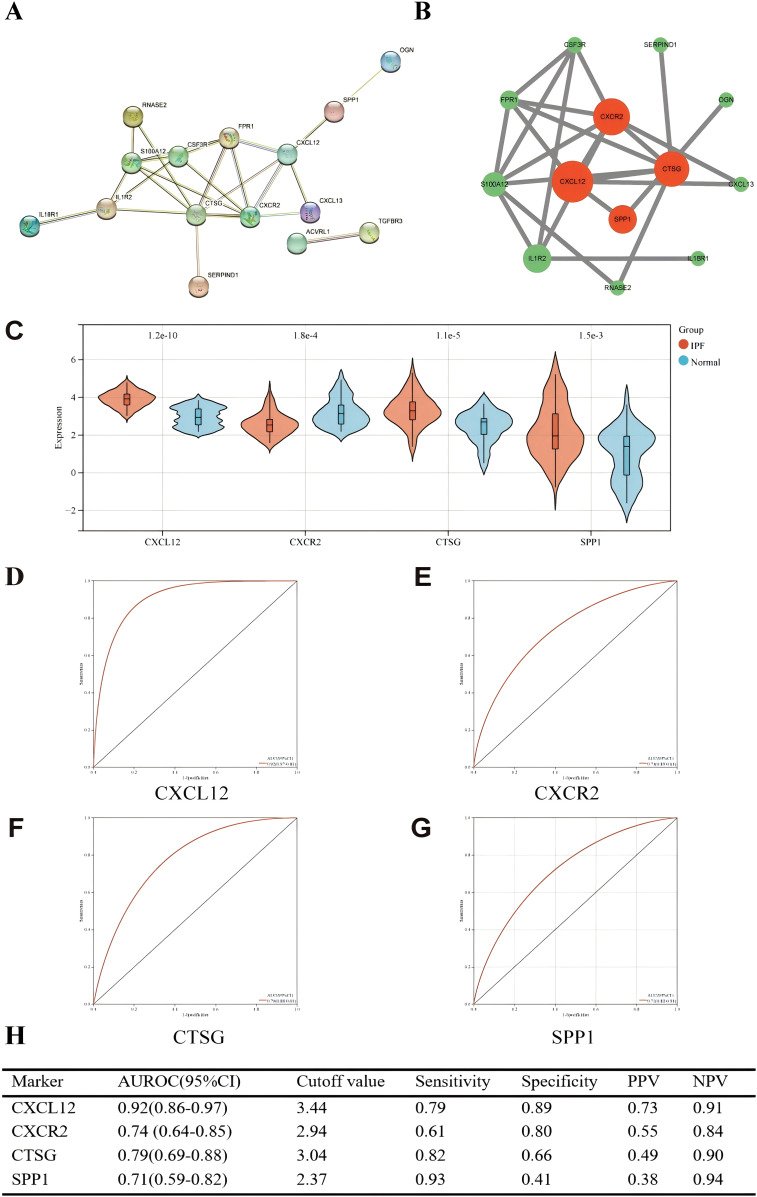
**(A)** PPI network showing the interactions of 15 immune-related genes. **(B)** Cytoscape analysis using the CytoNCA plugin identifying hub genes and their expression differences. **(C)** Expression differences of the four hub genes between IPF and control groups. **(D–G)** ROC curves of the four hub genes in IPF and control groups. **(H)** Diagnostic value of the four hub genes for differentiating between IPF and control groups. PPV, positive predictive value; NPV, negative predictive value; AUROC, area under the receiver operating characteristic curve.

We used the STRING online website for PPI network analysis. In total, a PPI network containing 15 nodes and 23 edges was obtained. Two of these genes lack association with other molecules and fail to form a molecular network. Altogether, a PPI network comprising 15 nodes and 23 edges was acquired (**Figure 10A**). The network was configured using the standard threshold (interaction score > 0.4). The Cytoscpe plugin CytoNCA was used to identify hub genes. Hub genes (*CXCL12*, *CXCR2*, *CTSG*, *SPP1*) were selected based on their top four scores (**Figure 10B**). **Figure 10C** shows the value of the four hub genes in the diagnosis of IPF in the training set. *CXCL12*, *CTSG*, and *SPP1* were significantly upregulated in the IPF group, while *CXCR2* was highly expressed in the normal group. Moreover, the AUROC values were 0.92 (95% CI = 0.86–0.97) (**Figures 10D, H**), 0.74 (95% CI = 0.64–0.85) (**Figures 10E, H**), 0.79 (95% CI = 0.70–0.88) (**Figures 10F, H**), and 0.71 (95% CI = 0.59–0.82) (**Figures 10G, H**). *CXCL12* emerged as the gene most strongly associated with IPF.

**Figure 11:**
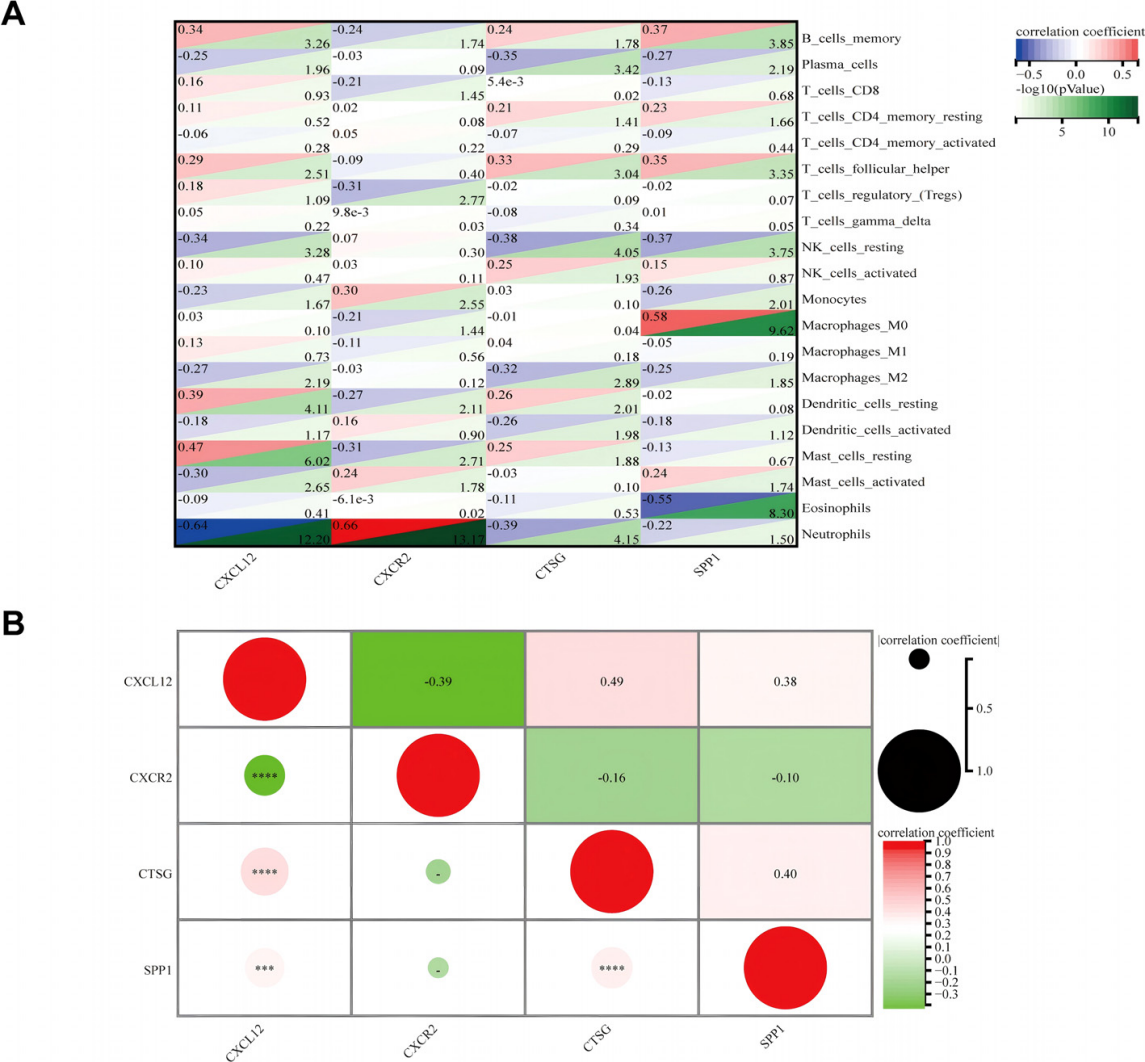
**(A)** Matrix correlation between immune-related hub genes and immune cell abundance. **(B)** Correlation analysis between the four hub genes. ^***^ *p* < 0.001; ^****^ *p* < 0.0001.

The correlation between four hub genes and immune cells is shown in **Figure 11A**. *CXCL12* was significantly correlated with five cell types, including a significant negative correlation with neutrophils (*r* = − 0.64, *p* < 0.001) and a significant positive correlation with resting mast (*r* = 0.47, *p* < 0.001). The only notable association observed between *CXCR2* and neutrophils was (*r* = 0.66, *p* < 0.001). There was an inverse relationship between *CTSG* and three types of immune cells, most notably with neutrophils (*r* = − 0.39, *p* < 0.001), and it was only positively correlated with follicular helper T (*r* = 0.33, *p* < 0.001). *SPP1* was significantly positively correlated with three immune cells and negatively correlated with two immune cells. The strongest correlation was with Macrophages_M0 (*r* = 0.58, *p* < 0.001). Meanwhile, we found a mild correlation between these four genes (**Figure 11B**).

**Figure 12:**
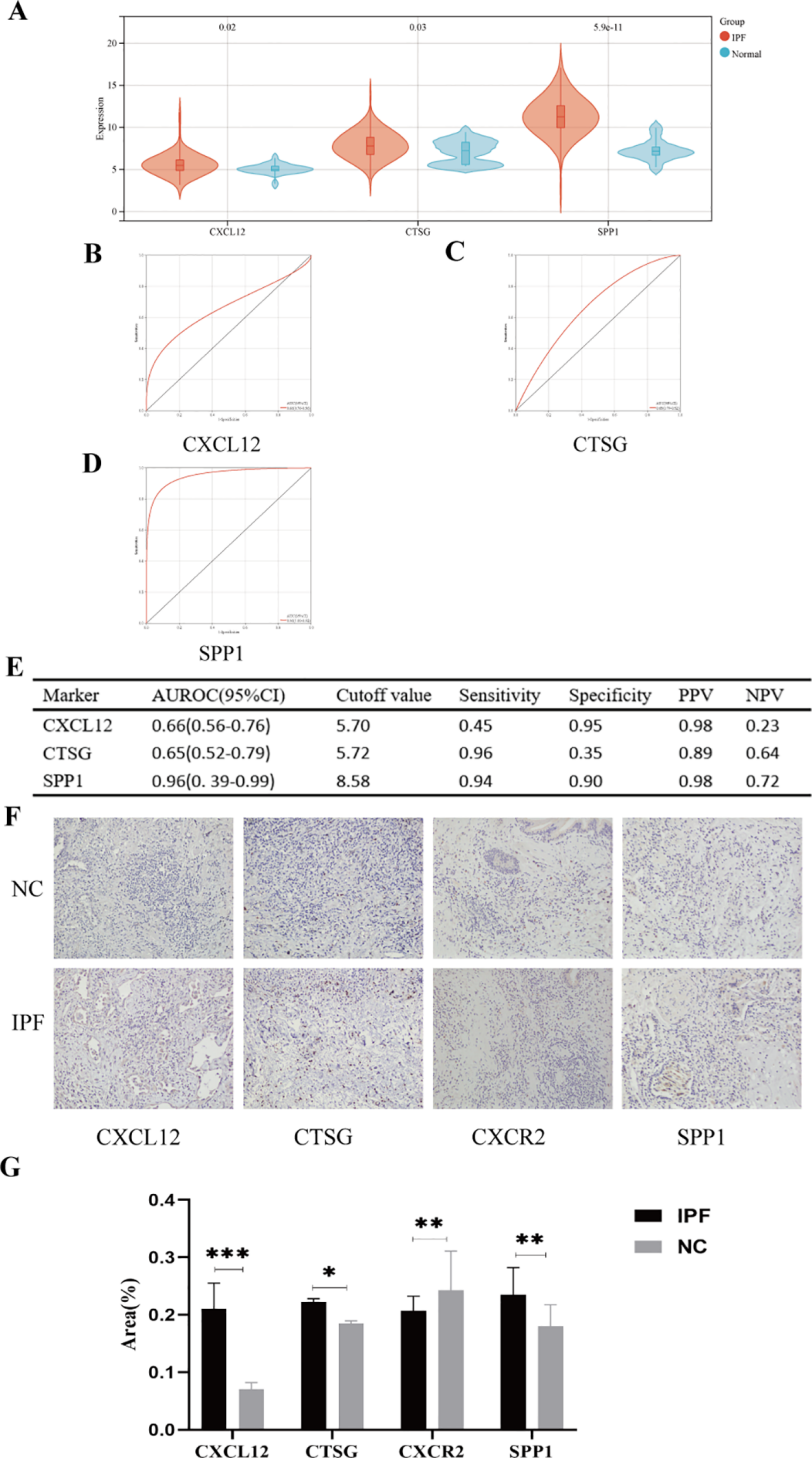
**(A)** Expression differences of the five hub genes between IPF and control groups. **(B–E)** ROC curves of the four hub genes in IPF and control groups. **(F)** Representative images of immunohistochemical of *CXCL12*, *CTSG*, *CXCR2*, and *SPP1* in lung tissues. **(G)** The areas of the four hub genes. PPV, positive predictive value; NPV, negative predictive value; AUROC, area under the receiver operating characteristic curve. ^*^ *p* < 0.05; ^**^ *p* < 0.01; ^***^ *p* < 0.001.

In the validation set (GSE70866), the expression levels of genes *CXCL12*, *CTSG*, and *SPP1* were consistent with those in the experimental set (**Figure 12A**); however, we regretfully were unable to detect the expression of *CXCR2*. The diagnostic efficacy of the three genes also yielded excellent results. The AUROC of *CXCL12* was 0.66 (95% CI = 0.56–0.76), with sensitivity and specificity of 0.45 and 0.95, respectively (**Figures 12B, E**). For *CTSG*, the AUROC was 0.65 (95% CI = 0.52–0.79), and the sensitivity and specificity were 0.96 and 0.35 (**Figures 12C, E**). The AUROC of *SPP1* in the diagnosis of IPF was 0.96 (95% CI = 0.39–0.99), and the sensitivity and specificity were 0.94 and 0.90, respectively (**Figures 12D, E**). We employed immunohistochemical methods to examine the expression profiles of four genes in both IPF and normal tissues. The results revealed that *CXCL12*, *CTSG*, and *SPP1* were significantly upregulated in IPF tissues, whereas *CXCR2* showed higher expression in normal tissues, which is consistent with our analytical findings (**Figures 12F, G**).

### Prediction of expression of immune-related hub genes in single-cell RNA-seq profiling

**Figure 13:**
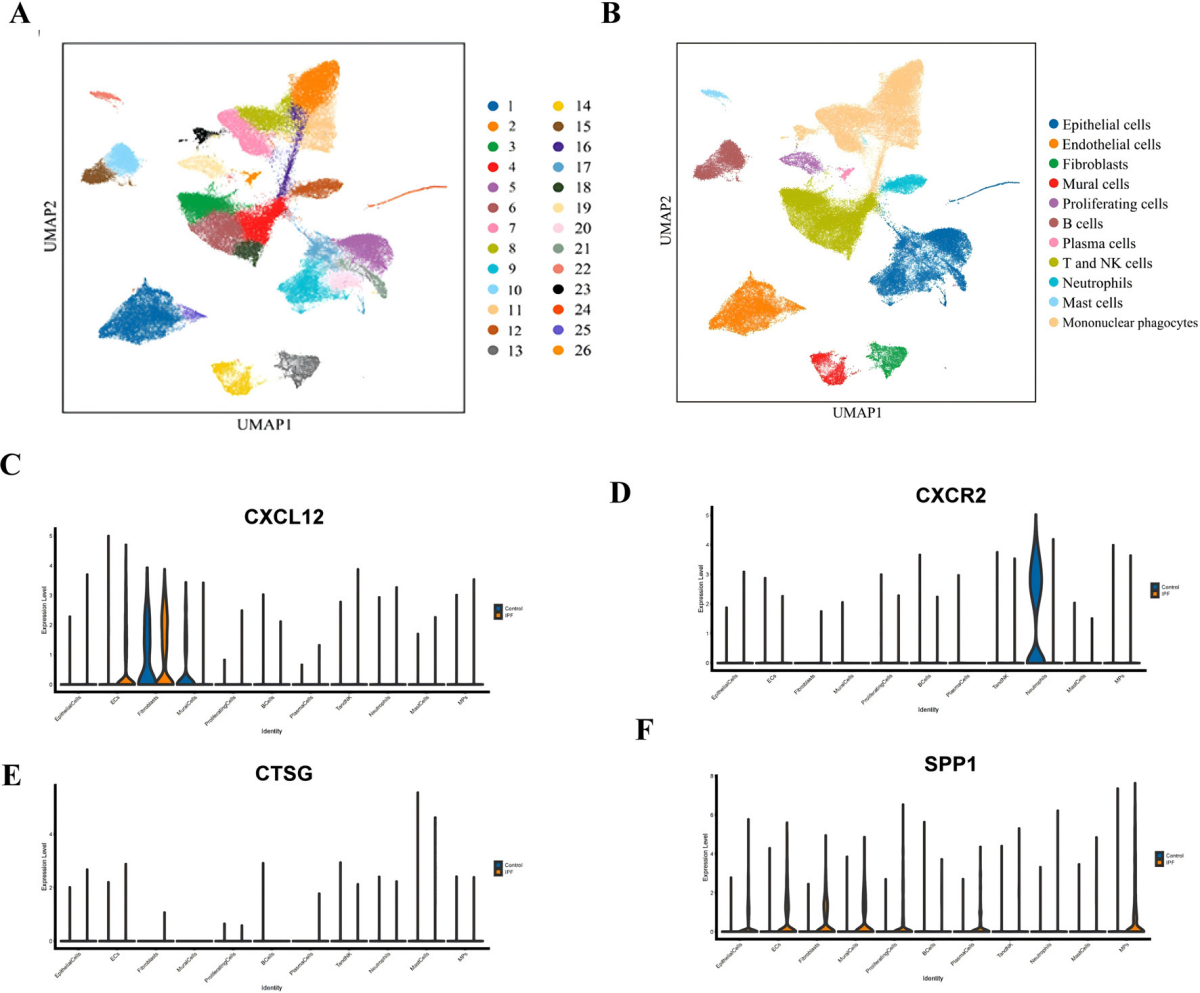
**(A)** UMAP plot of all cells from 26 cell clusters. **(B)** UMAP plot displaying the cell types in the two groups. **(C)** Gene expression of CXCL12 in IPF and normal groups. **(D)** Gene expression of CXCR2 in IPF and normal groups. **(E)** Gene expression of CTSG in IPF and normal groups. **(F)** Gene expression of *SPP1* in IPF and normal groups.

In this study, a total of eight lung tissue specimens were collected, comprising five cases diagnosed with IPF who underwent lung transplantation and three cases that underwent pulmonary nodule surgery but were ultimately determined to be benign. After sequencing the lung tissue cell suspensions using the 10× Genomics platform, a total of 90,722 single-cell high-quality data points were obtained. Following quality control and filtering, 75,613 cells were utilized for subsequent analysis (**Table 1**). After accounting for batch effects across samples, 26 distinct cellular populations were identified (**Figure 13A**). Based on previous research on canonical cell markers, these 26 cell clusters were categorized into 11 distinct cell types (**Figure 13B**). Including epithelial cells, endothelial cell subsets (ECs), fibroblasts, mural cells, prolific cells, B cells, plasma cells, T and NK cells, neutrophils, mast cells, and mononuclear phagocytes (MPs). **Figures 13C–F** depict the expression of immune-related hub genes across various specimens, with cell populations represented through a violin plot.

**Table 1:** Number and proportion of different cell types.

	IPF		Control	
Celltype	Numbers	Percentage	Numbers	Percentage
Epithelial cells	10321	23.53%	3471	10.93%
Endothelial cells	6902	15.74%	2552	8.04%
Fibroblasts	2015	4.59%	131	0.41%
Mural cells	1608	3.67%	477	1.50%
Proliferating cells	1047	2.39%	326	1.03%
Bcells	969	2.21%	4348	13.69%
Plasma cells	185	0.42%	294	0.93%
Tand NKcells	10506	23.96%	6856	21.59%
Neutrophils	1086	2.48%	1292	4.07%
Mast cells	348	0.79%	555	1.75%
Mononuclear phagocytes	8870	20.22%	11454	36.07%
Sum	43857	100%	31756	100%

## Discussion

IPF is a chronic, progressive, and devastating disease, primarily characterized by fibrosis, structural deformation, honeycomb lungs, fibrosis of the lamellar lung parenchyma, and the generation of fibroblastic foci, leading to poor prognosis and shorter survival (23). The incidence of IPF has risen in the past few years, with over 5,000 new confirmed cases annually in the UK (24). Aging is a major risk factor for IPF, with incidence rates doubling every decade after the age of 50 (4). Currently, there are no clinically effective treatments to stop or reverse pulmonary fibrosis (25). Most patients experience a gradual decline in lung function, which ultimately leads to severe respiratory failure and can be fatal (1). Due to the variability and uncertainty of IPF in the clinical process, early assessment and intervention of disease progression are particularly important.

Almost all cell types require copper for a multitude of physiological processes, and maintaining the equilibrium of copper in cells is critical for cellular physiology and endurance (26). Abnormalities in copper metabolism, as a novel mode of cell death, are closely linked to the emergence of numerous human illnesses (27–29). In addition, research has indicated that certain fibrotic conditions may be influenced by the levels of copper ions. One reason is that elevated copper ions in cells activate lysine oxidase, enhancing the cross-linking of collagen and elastin (30, 31). Therefore, this study links cuproptosis to the pathogenesis of IPF, with bioinformatics analysis identifying potential crucial genes and investigating prospective treatment targets.

In this study, we investigated the gene expression levels in the normal group and IPF patients using the GEO database, identifying a total of 92 DEGs. GO and KEGG enrichment analyses revealed enrichment in immune system processes, the extracellular matrix, fibronectin binding, transforming growth factor beta-activated receptor activity, and the cytokine–cytokine receptor interaction pathway. In addition, there was a notable difference in the percentage of immune cells between the IPF-affected group and the normal group. Our study showed that six immune cell markers—memory B cells, follicular helper T cells, activated NK cells, M1 macrophages, resting dendritic cells, and resting mast cells—were significantly highly expressed in the IPF group. Consistent with previous research, the expression of memory B cells, follicular T cells, and mast cells was in agreement with the study (32). However, the role of M1 macrophages in the pathogenesis of IPF remains controversial. Our results support the involvement of M1 macrophages in fibrosis. The mechanisms of action of activated NK cells and dormant DC cells in IPF were not identified and warrant further exploration.

Using bioinformatics analysis, we identified five cuproptosis-related hub genes that showed significant differences between the IPF and healthy groups, demonstrating high diagnostic efficacy. These results were validated in the Alveolar Irrigation Validation (GSE70866), further supporting the reliability of our findings.

*CFH* primarily inhibits the complement substitution pathways by accelerating the attenuation of the complement alternative pathway, specifically the C3-converting enzyme C3bBb, and impairing the production of novel C3b (33). Furthermore, C3b interacts with other proteins to promote the restoration of normal immune system functioning (34). Previous studies have shown that immune disorders drive the pathophysiology of IPF (35). In our study, *CFH* was highly expressed in the tissues of IPF patients, suggesting it may act as a potential disruptor. These findings lead us to hypothesize that *CFH* could play a role in IPF as an immune system suppressor. Previous studies have shown that *STEAP1* expression is upregulated in lung adenocarcinoma cells, where it regulates cellular epithelial–mesenchymal transition (EMT) through the (JAK2/STAT3) signaling pathway (36). In addition, studies have shown that *STEAP1* is closely linked to abnormal copper metabolism (37). However, it is still unclear whether abnormal copper metabolism in epithelial cells plays a role in mediating EMT. Similarly, in lung adenocarcinoma research, factors derived from *HDC* + pmn-mdscs may influence EMT cell behavior through paracrine models. Blocking these factors inhibits metastasis in lung adenocarcinoma (38). The study suggests that *HDC* knockout improves the progression of liver fibrosis (39). However, no related literature currently exists regarding IPF, which may offer new directions for future research. *NUDT16* is a (deoxygenated) creatine diphosphatase, primarily responsible for protecting cells from the harmful effects of inosine triphosphate (ITP) in the nucleus. The ITP receptor facilitates the transformation of lung fibroblasts into myofibroblasts, potentially contributing to lung fibrosis (40). Our results suggest that *NUDT16* is poorly expressed in IPF patients, indicating that *NUDT16* may act as a protective factor in the mechanisms of IPF. *FMO5*, a member of the *FMO* protein family, is involved in the upregulation of the *NRF2*-mediated oxidative stress response (41). *NRF2* has been shown to attenuate renal fibrosis through the PI3K/AKT signaling pathway (42). Additionally, *NRF2* activation has been demonstrated to protect against various lung diseases, including IPF (43). This validates our findings. Furthermore, we analyzed the correlation among these five genes and identified significant synergistic or antagonistic interactions between them.

All stages of IPF involve both innate and adaptive immune responses (21). IRGs are essential for immune cells to respond to immune stimulation and infiltration (44). However, the regulatory mechanism of IPF through IRG expression remains unclear. From the IMMport database, we identified 1,793 IRGs and intersected them with previously identified DEGs, resulting in 24 DEIRGs. KEGG analysis revealed that these DEIRGs are primarily associated with the cytokine–cytokine receptor interaction pathway. GO enrichment analysis indicated the following: defense response, immune system process, and regulation of immune system process (biological processes); extracellular (cellular composition); and enrichment of cytokine binding (molecular function). These findings align with our initial hypothesis. Subsequently, four hub genes (*CXCL12*, *CXCR2*, *CTSG*, and *SPP1*) were identified in this study using PPI network analysis. Among these, *CXCL12*, *CTSG*, and *SPP1* were highly expressed in IPF, while *CXCR2* was expressed at lower levels, consistent with immunohistochemistry results. Similarly, the AUROC analysis showed a moderate diagnostic capability for these genes.

*CXCL12* has garnered widespread attention as the sole ligand of *CXCR4*, a major chemokine receptor on fibroblasts (45). Research indicates that the *CXCL12-CXCR4* axis plays a role in multiple pathological processes of fibrosis, including inflammation, immune responses, epithelial–mesenchymal transitions, and the formation of new blood vessels (46). Our bioinformatics analysis found that *CXCL12* expression was upregulated in IPF patients, a finding that was validated by previous studies (47). In our single-cell sequencing, we found that *CXCL12* is predominantly enriched in ECs, providing new evidence for exploring IPF. *CTSG* is a protein-coding gene, and single-cell sequencing showed statistically significant differences in CTSG expression, primarily in monocyte macrophages, which regulate damage and repair in various fibrosis models. A recent study showed that macrophages from both mice and humans promote fibrosis by overexpressing repair mechanisms in alveolar damage (48). The study (49) suggests that *CTSG* is upregulated in the peripheral blood of IPF patients, consistent with our findings. Previous research (50) reports that macrophages orchestrate fibroblast activation via *Spp1*, *Fn1*, and *Sema3* crosstalk. *SPP1* has been found to play an important role in fibrosis in multiple organs, including the heart, lungs, and skin (51, 52). Our single-cell sequencing revealed significant differences in *SPP1* expression in multiple cell types, including ECS, EPI, and NK, with the largest differences observed in EC subsets. Therefore, we hypothesize that *SPP1* may regulate fibrosis not only through macrophages but also through ECs, providing a new direction for subsequent analysis. *CXCR2* is a receptor for interleukin 8 (IL-8), which mediates neutrophil migration to inflammatory sites (53). Our bioinformatics analysis results suggest that *CXCR2* is highly expressed in normal tissues, and single-cell sequencing results further confirm that this high expression is predominantly concentrated in neutrophils. In contrast to some of the current findings (54), we suggest that the high expression of *CXCR2* in normal tissues stimulates the recruitment of more neutrophils, rapidly suppresses inflammation, prevents excessive repair of postinflammatory tissue, and thus reduces fibrosis. However, this is merely a hypothesis, and further research is needed to confirm it.

Additionally, we examined the relationship between four specific genes and immune cells. *CXCL12* was significantly negatively associated with neutrophils, while *CTSG* and *SPP1* were significantly positively correlated with resting mast cells and macrophages, respectively. *CXCR2* was only significantly positively associated with neutrophils, which is consistent with our single-cell sequencing results. A moderate correlation was observed between the four genes. These findings suggest an important association between IPF and IRGs and may provide novel perspectives and directions for future research.

This study has some limitations. First, databases of genes associated with CRGs and IRGs are limited, and more data need to be mined. Second, the single-cell sequencing results are based on single-center clinical specimens with a limited sample size, and additional cases may be needed for validation due to patient heterogeneity.

## Conclusion

This study confirms that the development of IPF may be associated with cuproptosis and identifies five key related genes (*CFH*, *STEAP1*, *HDC*, *NUDT16*, and *FMO5*). Additionally, we found a strong relationship between IPF and immune cells, leading to the identification of four important genes (*FMO5*, *CFH*, *HDC*, and *STEAP1*). Single-cell sequencing results further elucidated their expression in relevant cell clusters. Therefore, these selected genes may serve as potential biomarkers and therapeutic targets for future IPF research.

## Data Availability

The data supporting the findings of this study are deposited in GEO (http://www.ncbi.nlm.nih.gov/geo), GeneCard (https://www.genecards.org), ImmPort (https://www.immport.org/shared/genelists) database.
